# Neoadjuvant chemotherapy versus surgery first for resectable pancreatic cancer (Norwegian Pancreatic Cancer Trial - 1 (NorPACT-1)) – study protocol for a national multicentre randomized controlled trial

**DOI:** 10.1186/s12893-017-0291-1

**Published:** 2017-08-25

**Authors:** Knut Jørgen Labori, Kristoffer Lassen, Dag Hoem, Jon Erik Grønbech, Jon Arne Søreide, Kim Mortensen, Rune Smaaland, Halfdan Sorbye, Caroline Verbeke, Svein Dueland

**Affiliations:** 1 Department of Hepato-Pancreato-Biliary Surgery, Oslo University Hospital, Oslo, Norway; 2 Department of Acute and Digestive Surgery, Haukeland University Hospital, Bergen, Norway; 3 Department of Gastrointestinal Surgery, St. Olavs Hospital, Trondheim University Hospital, Trondheim, Norway; 4 Department of Clinical and Molecular Medicine, Norwegian University of Science and Technology, Trondheim, Norway; 5 Department of Gastrointestinal Surgery, Stavanger University Hospital, Stavanger, Norway; 6 Department of Clinical Medicine, University of Bergen, Bergen, Norway; 7 Department of Gastrointestinal and Hepatobiliary Surgery, University Hospital of Northern Norway, Tromsø, Norway; 8 Department of Haematology and Oncology, Stavanger University Hospital, Stavanger, Norway; 9 Department of Oncology, Haukeland University Hospital, Bergen, Norway; 10 Department of Clinical Science, Haukeland University Hospital, University of Bergen, Bergen, Norway; 11 Department of Pathology, Oslo University Hospital, Oslo, Norway; 12 Institute of Clinical Medicine, University of Oslo, Oslo, Norway; 13 Department of Oncology, Oslo University Hospital, Oslo, Norway

**Keywords:** Resectable pancreatic cancer, Neoadjuvant chemotherapy, Overall survival

## Abstract

**Background:**

Pancreatic cancer is the fourth leading cause of cancer-related death. While surgical resection remains the foundation for potentially curative treatment, survival benefit is achieved with adjuvant oncological treatment. Thus, completion of multimodality treatment (surgical resection and (neo)adjuvant chemotherapy) to all patients and early treatment of micrometastatic disease is the ideal goal. NorPACT–1 aims to test the hypothesis that overall mortality at one year after allocation of treatment can be reduced with neoadjuvant chemotherapy in surgically treated patients with resectable pancreatic cancer.

**Methods/Design:**

The NorPACT– 1 is a multicentre, randomized controlled phase III trial organized by the Norwegian Gastrointestinal Cancer Group for Hepato-Pancreato-Biliary cancer. Patients with resectable adenocarcinoma of the pancreatic head are randomized to receive either surgery first (Group 1: SF/control) or neoadjuvant chemotherapy (Group 2: NT/intervention) with four cycles FOLFIRINOX followed by resection. Both groups receive adjuvant chemotherapy with gemicitabine and capecitabine (six cycles in Group 1, four cycles in Group 2). In total 90 patients will be randomized in all the five Norwegian university hospitals performing pancreatic surgery. Primary endpoint is overall mortality at one year following commencement of treatment for those who ultimately undergo resection. Secondary endpoints are overall survival after date of randomization (intention to treat), overall survival after resection, disease-free survival, histopathological response, complication rates after surgery, feasibility of neoadjuvant and adjuvant chemotherapy, completion rates of all parts of multimodal treatment, and quality-of-life. Bolt-on to the study is a translational research program that aims at identifying factors that are predictive of response to NT, the risk of distant cancer spread, and patient outcome.

**Discussion:**

NorPACT– 1 is designed to investigate the additional benefit of NT compared to standard treatment only (surgery + adjuvant chemotherapy) for resectable cancer of the pancreatic head to decrease early mortality (within one year) in resected patients.

**Trial registration:**

Trial open for accrual 01.02.2017.

ClinicalTrials.gov Identifier: NCT02919787. Date of registration: September 14, 2016.

## Background

Pancreatic cancer is the fourth leading cause of cancer-related death in Europe and the United States [1, 2]. Surgical resection remains the only potentially curative treatment. However, the median survival of patients undergoing pancreatic resection alone is 16–23 months, with a 5-year overall survival between 10 and 20% [3–6]. Adjuvant chemotherapy improves the median and 5-year overall survival [4, 7]. Thus, completion of multimodality treatment is the ideal goal and standard of care for treatment of pancreatic ductal adenocarcinoma (PDAC). It is well known that initiation and completion of adjuvant chemotherapy can be precluded by perioperative complications [6, 8, 9]. Complications following pancreatoduodenectomy are encountered in 40–50% of patients, with a perioperative mortality rate of 2–4% [10–12]. The technical complexity of the operation and the frailty and co-morbidity of the patient population contribute to the high rate of complications. A significant proportion of patients undergoing pancreatectomy for PDAC develops recurrent disease within 2 years after surgery, and about 20% of the patients have early disease progression within six months after resection [6, 13]. Likely, patients with early distant recurrence had occult metastasis at the time of operation, and may thus have been inadequately selected for surgery. However, useful clinical criteria for accurate prediction of patients suspectable to suffer an early distant or loco-regional recurrence are not available.

Currently, the surgery-first (SF) strategy is the most universally accepted approach (and the standard of care in Norway) to the treatment of resectable PDAC. Still, the optimal sequence of surgery and chemotherapy remains unclear [14, 15]. In three European well-designed randomized controlled trials that accrued patients with good performance status and following stringent tumour biology criteria (such as low CA 19–9 levels), the initiation rate of adjuvant therapy was 83–90% [4, 5, 7]. However, only 50–62% completed multimodal treatment in these highly selected patients. Given the significant survival benefit of adjuvant chemotherapy, the completion rates reported in the literature remain too low. Some centres advocate neoadjuvant chemotherapy (NT) as an alternative to the SF approach [16, 17]. Proponents of the NT strategy suggest that the negative impact of early cancer progression and postoperative complications upon completion of multimodality treatment is reduced by delivery of NT prior to pancreatoduodenectomy [8]. However, the scheduled resection has to be cancelled in up to 20% of patients receiving NT due to early metastases, reduced performance-status or comorbidities during NT, but very rarely due to local tumour progression alone [17]. Chemotherapy employed upfront (before surgery) in patients with resectable pancreatic cancer could potentially increase the proportion of patients who eventually received both treatment modalities, and thus, may benefit from a combined effect.

To date, there are no prospective data proving the superiority of one sequence strategy over the other. Recent studies have shown promising results using NT with FOLFIRINOX for locally advanced and borderline resectable pancreatic cancer, and for the palliative treatment of metastatic pancreatic cancer [18, 19]. Available data on early distant recurrence after pancreatoduodenectomy or during NT support the concept of pancreatic cancer as a systemic disease, even in early-stage settings [20]. Currently, by comparing multi-agent regimens to single-agent approaches efforts are made to bring systemic therapy upfront to study the effect of aggressive chemotherapy regimens in well-designed clinical trials. To circumvent patient selection bias, only a randomized comparison can objectively show benefit of one strategy over the other. The purpose of this study is to further investigate the benefit of adding NT in comparison to standard treatment only for resectable cancer of the pancreatic head (surgery followed by adjuvant chemotherapy).

## Methods/Design

### Design

**Fig. 1:**
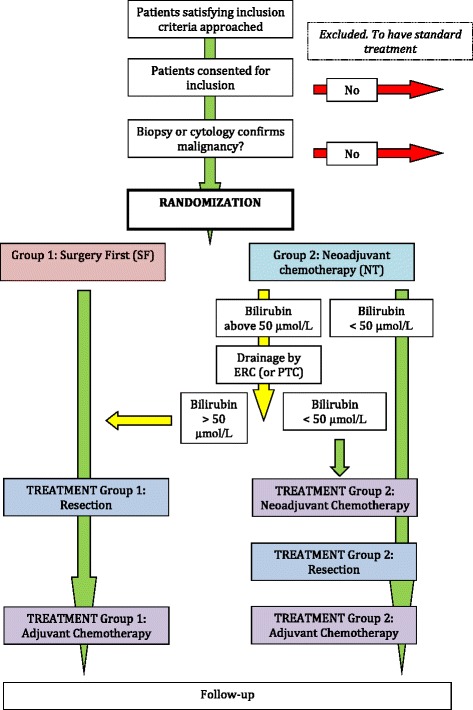
Flowchart of the Norwegian pancreatic cancer trial-1

The Norwegian Pancreatic Cancer Trial (NorPACT) - 1 is a multicentre, randomized controlled phase III trial organized by the Norwegian Gastrointestinal Cancer Group (NGICG) for Hepato-Pancreato-Biliary (HPB) cancer. Eligible patients are randomized in non-equal groups (3:2) to either receive NT followed by resection or standard treatment ((pancreatoduodenectomy) followed by adjuvant chemotherapy) (Fig. 1). The aim of this study is to evaluate the additional benefit of NT to the standard treatment (surgery + adjuvant chemotherapy) to decrease early mortality (within one year) in resected patients with resectable cancer of the pancreatic head.

#### Primary end points:

- □ overall mortality at one year following commencement of treatment (NT or SF) for those patients who undergo resection (i.e. only resected patients are included in the analysis)

#### Secondary end points:

- □ overall survival after date of randomization (intention to treat)
- □ overall survival following resection
- □ overall survival after 3 and 5 years
- □ disease-free survival
- □ histopathological tumour stage ((y)pTN), R0 rate, grade of tumour regression
- □ complication rates after surgery (30 and 90 days, Dindo-Clavien and International Study Group of Pancreatic Surgery (ISGPS) classification systems) [21–25]
- □ feasibility of neoadjuvant and adjuvant chemotherapy (Common Terminology Criteria for Adverse Events, grade 3–5, dose reduction, dose delay)
- □ completion rates of all parts of multimodal treatment
- □ quality of life (EORTC QLQ-30)
- □ performance status (ECOG) compared to baseline values
- □ exploratory translational research

### Study population

Patients meeting the National Comprehensive Cancer Network criteria for resectable pancreatic adenocarcinoma in the pancreatic head are eligible [14]. This implies: 1) no tumour contact with the superior mesenteric vein or portal vein or ≤180 ° contact without vein contour irregularity, 2) no arterial tumour contact (coeliac axis, common hepatic artery or superior mesenteric artery), 3) no distant metastasis.

#### Inclusion criteria (all of the following):

- □ resectable ductal adenocarcinoma of the pancreatic head
- □ T1–3, Nx, M0 (UICC 7th edition, 2010)
- □ cytological or histological confirmation or strong suspicion of adenocarcinoma
- □ age > 18 year and considered fit for major surgery
- □ written informed consent
- □ considered able to receive the study-specific chemotherapy

#### Exclusion criteria (one or more of the following):

- □ co-morbidity precluding pancreatoduodenectomy
- □ histological type other than ductal adenocarcinoma
- □ chronic neuropathy ≥ grade 2
- □ World Health Organization performance score > 2
- □ granulocyte count <1500 per cubic millimetre
- □ platelet count <100,000 per cubic millimetre
- □ serum creatinine >1.5 UNL (upper limit normal range)
- □ albumin <2.5 g/dl
- □ female patients in child-bearing age not using adequate contraception, pregnant or lactating women
- □ mental or physical disorders that could interfere with treatment or the provision of informed consent
- □ other malignancy within the past 5 years, except curatively treated non-melanomatous skin or non-invasive cervical cancer
- □ percutaneous tumour biopsy
- □ any reason why, in the opinion of the investigator, the patient should not participate

### Locations

Surgery for malignancies of the pancreatic head is currently performed only at five university hospitals in Norway. These departments have all agreed to participate in this trial.

Participating centres are: Oslo University Hospital, Haukeland University Hospital (Bergen), Stavanger University Hospital, St. Olav University Hospital (Trondheim), and the University Hospital Northern Norway in Tromsø. Each centre has a main investigator who liaises with the central study board.

### Time frame

In Norway, annually 60–70 patients are expected to have primary resectable pancreatic adenocarcinoma in the pancreatic head. Patients will be recruited into the trial between 1st of January 2017 and 31st of December 2019 (36 months).

### Randomization

After the patient has given oral and written consent, computer-generated randomization will be performed. Randomization is to either

- □ GROUP 1 (control): Surgery First
- □ GROUP 2 (intervention): Neoadjuvant Chemotherapy

There will be an overweight of Group 2 allocations by a ratio of 3:2 to ensure equal groups at primary endpoint (cfr. below). Randomization is stratified for each centre and will be generated in blocks with unknown and varying size (4–6 patients per block) to ensure that groups are balanced at all centres, irrespective of the final number of patients recruited.

### Sample-size calculation

Based on available data, we assume a one-year overall mortality rate of 25% in the SF arm (Group 1) [5, 6, 26]. Based on highly selected patient series, a suggested one-year mortality rate of 5% is estimated in patients who receive NT and eventually a pancreatoduodenectomy (Group 2) [16, 17]. We aim to evaluate if this improvement is achievable in a randomized controlled trial. To show a reduction in one-year mortality rate from 25 to 5% in a two-armed, parallel-group design with alpha (significance level) 0.05 and beta (power) of 0.79, a sample of 34 patients per group is needed.

An estimated one-third of the NT group will not reach resection and thus not be available for evaluation with regard to the primary endpoint. Furthermore, we estimate that two patients per study group will have their scheduled surgery aborted because of unexpected intraoperative findings. To correct for these patients, a 3:2 randomization is designed, with 36 patients randomized to SF (Group 1) and 54 patients to NT (Group 2), yielding a total of 90 patients.

### Handling cross-over, drop-outs and exclusion

Post-randomisation exclusion is to be avoided at “all costs”. While patients are offered to withdraw without giving any reason, this is not considered very likely as both groups provide standard treatment of today, albeit at reversed sequence for some (Group 2). Patients who for some reason cannot fulfil neoadjuvant chemotherapy (Group 2) but are still considered candidates for resection will be offered resection according to standard criteria. The reasons for this might be:

- Inability to achieve adequate reduction of bilirubin levels by drainage within 4 weeks
- Massive adverse reactions to first cycle chemotherapy

These patients are considered cross-over provided no single cycle was completed, but remain under analysis by intention-to-treat. They are not excluded from the trial. Patients who suffer other incidents post-randomisation but prior to any treatment are still analysed under intention-to-treat.

### Treatment

#### Chemotherapy

##### Neoadjuvant group (Group 2)

Neoadjuvant chemotherapy consists of 4 cycles (2 months) of FOLFIRINOX (oxaliplatin 85 mg/m^2^, irinotecan 180 mg/m^2^, leucovorin 400 mg/m^2^, and 5-fluorouracil (400 mg/m^2^ bolus then 2400 mg/m^2^ over 46 h)) [18, 19]. Patients who undergo surgical resection will receive adjuvant chemotherapy with 4 cycles (4 months) Gemicitabine 1000 mg/m^2^ over 30 min at day 1, 8, 15 of each 28-day cycle and capecitabine 830 mg/m^2^ ×2 daily for 3 weeks and one week rest of each 28-day cycle [27]. Adjuvant chemotherapy must be started within 12 weeks after resection [28]. Dose reduction or dose delays are acted upon according to local clinical practice at each centre.

##### Surgery-first group (Group 1)

Following pancreatic resection, all patients receive adjuvant chemotherapy with 6 cycles (6 months) Gemicitabine 1000 mg/m^2^ over 30 min at day 1, 8, 15 of each 28-day cycle and capecitabine 830 mg/m^2^ ×2 daily for 3 weeks and one week rest of each 28-day cycle [27]. Adjuvant chemotherapy must be started within 12 weeks after resection [28].

Side effects of chemotherapy are graded by the “Common Terminology Criteria for Adverse Events” version 4 https://evs.nci.nih.gov/ftp1/CTCAE/About.html. Grade 3–5 are reported. At each study visit, laboratory parameters are determined for dose adjustments. Dose reduction or dose delays are acted upon according to local clinical practice at each centre.

#### Surgery

Surgery is scheduled within 4 weeks in the control arm, and also within 4 weeks after the last neoadjuvant infusion in the treatment arm. Resection of the pancreatic head will be performed as a standard or pylorus-preserving pancreatoduodenectomy with standard lymphadenectomy [29]. The reconstruction is done by a retrocolic end-to-side pancreatico-jejunostomy and an end-to-side hepatico-jejunostomy. In addition, an end-to-side duodeno- or gastrojejunostomy is performed on a jejunal alpha-loop. Otherwise, each centre may use its standard perioperative management for pancreatoduodenectomies. Surgical morbidity is assessed by the Dindo/Clavien classification [21, 30]. Pancreatic fistulas, biliary leakages, delayed gastric emptying and postoperative haemorrhage are reported according to the definitions of the ISGPS [22–25].

### Pathology examination

The resection specimen will be evaluated according to a detailed standardized pathology examination protocol [31, 32]. At the discretion of the local team, the anterior and posterior surfaces of the pancreas, as well as the superior mesenteric vein groove and surface towards the superior mesenteric artery may be inked by the surgeon according to an agreed colour code. The histopathological response of the tumor to the neoadjuvant treatment will be assessed according to an established tumour regression grading system [33].

### Follow-up

Follow-up is based on physical examination, blood samples (hemoglobim, white blood cell count, differential blood count, platelets, creatinin, bilirubin, aspartate aminotransferase, alanine aminotransferase, lactate dehydrogenase, gamma-glutamyl transpeptidase, prothrombin time, albumin, sodium, potassium, CA19–9 and carcinoembryonic antigen) and CT scans of chest and abdomen at 6, 9, 12, 15 months after surgery and every six months thereafter until disease recurrence or, in patients without relapse, at 5-years following surgery. Any newly appearing lesion with histological documentation of cancer defines recurrent disease. Also, any newly appearing lesion(s) suspicious for malignancy without histological documentation but increasing in size upon repeated follow-up exams, especially in the context of progressive symptoms (pain, weight loss) or increasing tumor marker (CA 19–9) levels, are considered recurrent disease, either distant, regionally or in the former surgical bed. The date of recurrence is defined as the date of radiological or histological evidence of relapse. The study ends when the last randomized patient has been followed for 5 years after surgery.

### Quality of life

Quality of life will be assessed by the QLQ-30 of the European Organization for Research and Treatment of Cancer at study inclusion, before randomization, before surgery in the neoadjuvant arm, and at 4 weeks after surgery, as well as at any follow-up visit.

### Translational research

Blood samples and tumour tissue will be stored to enable further translational research.

### Safety

Suspected Unexpected Serious Adverse Reactions will be reported to the Competent Authority and Ethics Committee according to national regulation. The sponsor will ensure that all relevant information about suspected serious unexpected adverse reactions that are fatal or life-threatening is recorded and reported as soon as possible to the Competent Authority and Ethics Committee after knowledge by the sponsor of such a case, and that relevant follow-up information is subsequently communicated. All other suspected serious unexpected adverse reactions will be reported to the Competent Authority concerned and to the Ethics Committee concerned as soon as of first knowledge by the sponsor. A Data Monitoring Committee will be established with specialists in gastroenterological surgery and medical oncology. The study will be closed if 50% or more of the patients randomized to NT do not receive the planned surgery or if the reason for not receiving surgery is reduced performance status or side effects due to neoadjuvant chemotherapy in more than 30% of the randomized patients.

### Ethics

The study protocol was approved by the Regional Committee for Medical and Health Research Ethics (2015/610/REK Nord) and The Norwegian Medicines Agency (15/05308–8). Patients must provide written consent before entering the trial. All data will be handled with strict confidentiality, and study reports or presentations will maintain the anonymity of patients.

## Discussion

The NorPACT-1 study investigates the benefit of NT to the standard treatment (surgery + adjuvant chemotherapy) for resectable cancer of the pancreatic head as a means of avoiding early mortality (within one year) in resected patients. The study results will show which of the two treatment strategies is superior with respect to survival and quality of life in patients undergoing surgery for resectable pancreatic cancer. Non-randomized data have provided some support for NT, but these observations may be biased by patient selection bias. Hence, only a randomized comparison can objectively show benefit of one strategy over the other. The main rationale for NT in this patient group is twofold. First, upfront chemotherapy enable early treatment of micrometastases. Second, postoperative complications often preclude initiation or completion of adjuvant chemotherapy and thereby annul the benefits of multimodal treatment. By delivering NT prior to surgical resection, the chance of receiving both modalities is increased. Furthermore, NT is likely to result in improved patient selection for surgery, as individuals with rapidly progressive disease under NT can be spared from major surgery, which is unlikely to be beneficial and associated with significant morbidity risk. Systemic therapy in NorPACT-1 is given with chemotherapy combinations that have shown promising results in two recent randomized controlled trials [19, 27]. The multicentre design with participation of the five centres that perform pancreatic surgery in Norway ensures that any eligible patient has the opportunity to participate in the clinical trial at the centre closest to his/her home. Bolt-on to the proposed study is a translational research program that aims at identifying factors that are predictive of response to NT, the risk of distant cancer spread and patient outcome. These potential biomarkers will allow better patient selection for surgery and/or NT.

## Abbreviations

CA19–9: Cancer antigen 19–9
CT: Computer tomography
ECOG: Eastern cooperative oncology group
EORTC QLQ: European organization for research and treatment of cancer quality of life questionnaire
FOLFIRINOX: Oxaliplatin, irinotecan, fluorouracil, and leucovorin
ISGPS: International Study Group of Pancreatic Surgery
NGICG-HPB: Norwegian Gastrointestinal Cancer Group for Hepato-Pancreato-Biliary cancer
NorPACT-1: Norwegian Pancreatic Cancer Trial-1
NT: Neoadjuvant chemotherapy
PDAC: Pancreatic ductal adenocarcinoma
SF: Surgery first
TNM: Tumor, node metastasis staging system

## References

[1] Malvezzi M, Arfe A, Bertuccio P, Levi F, La VC, Negri E (2011). European cancer mortality predictions for the year 2011. Ann Oncol.

[2] Siegel R, Ma J, Zou Z, Jemal A (2014). Cancer statistics, 2014. CA Cancer J Clin.

[3] Winter JM, Brennan MF, Tang LH, D'Angelica MI, DeMatteo RP, Fong Y (2012). Survival after resection of pancreatic adenocarcinoma: results from a single institution over three decades. Ann Surg Oncol.

[4] Neoptolemos JP, Stocken DD, Friess H, Bassi C, Dunn JA, Hickey H (2004). A randomized trial of chemoradiotherapy and chemotherapy after resection of pancreatic cancer. N Engl J Med.

[5] Neoptolemos JP, Stocken DD, Bassi C, Ghaneh P, Cunningham D, Goldstein D (2010). Adjuvant chemotherapy with fluorouracil plus folinic acid vs gemcitabine following pancreatic cancer resection: a randomized controlled trial. JAMA.

[6] Labori KJ, Katz MH, Tzeng CW, Bjornbeth BA, Cvancarova M, Edwin B (2016). Impact of early disease progression and surgical complications on adjuvant chemotherapy completion rates and survival in patients undergoing the surgery first approach for resectable pancreatic ductal adenocarcinoma - a population-based cohort study. Acta Oncol.

[7] Oettle H, Post S, Neuhaus P, Gellert K, Langrehr J, Ridwelski K (2007). Adjuvant chemotherapy with gemcitabine vs observation in patients undergoing curative-intent resection of pancreatic cancer: a randomized controlled trial. JAMA.

[8] Tzeng CW, Tran Cao HS, Lee JE, Pisters PW, Varadhachary GR, Wolff RA (2014). Treatment sequencing for resectable pancreatic cancer: influence of early metastases and surgical complications on multimodality therapy completion and survival. J Gastrointest Surg.

[9] Merkow RP, Bilimoria KY, Tomlinson JS, Paruch JL, Fleming JB, Talamonti MS (2014). Postoperative complications reduce adjuvant chemotherapy use in resectable pancreatic cancer. Ann Surg.

[10] Ziegler KM, Nakeeb A, Pitt HA, Schmidt CM, Bishop SN, Moreno J (2010). Pancreatic surgery: evolution at a high-volume center. Surgery.

[11] Winter JM, Cameron JL, Campbell KA, Arnold MA, Chang DC, Coleman J (2006). 1423 pancreaticoduodenectomies for pancreatic cancer: a single-institution experience. J Gastrointest Surg.

[12] Gouma DJ, van Geenen RC, van Gulik TM, de Haan RJ, de Wit LT, Busch OR (2000). Rates of complications and death after pancreaticoduodenectomy: risk factors and the impact of hospital volume. Ann Surg.

[13] Groot VP, Rezaee N, Wu W, Cameron JL, Fishman EK, Hruban RH, et al. Patterns, timing, and predictors of recurrence following pancreatectomy for pancreatic ductal adenocarcinoma. Ann Surg. 2017. [Epub ahead of print]. 10.1097/SLA.0000000000002234 28338509

[14] National Comprehensive Cancer Network Clinical Practice Guidelines in Oncology: Pancreatic adenocarcinoma v2.2015. 2015. Accessed 31 Dec 2016.

[15] Ducreux M, Cuhna AS, Caramella C, Hollebecque A, Burtin P, Goere D (2015). Cancer of the pancreas: ESMO clinical practice guidelines for diagnosis, treatment and follow-up. Ann Oncol.

[16] Christians KK, Heimler JW, George B, Ritch PS, Erickson BA, Johnston F (2016). Survival of patients with resectable pancreatic cancer who received neoadjuvant therapy. Surgery.

[17] Tzeng CW, Fleming JB, Lee JE, Xiao L, Pisters PW, Vauthey JN (2012). Defined clinical classifications are associated with outcome of patients with anatomically resectable pancreatic adenocarcinoma treated with neoadjuvant therapy. Ann Surg Oncol.

[18] Ferrone CR, Marchegiani G, Hong TS, Ryan DP, Deshpande V, McDonnell EI (2015). Radiological and surgical implications of neoadjuvant treatment with FOLFIRINOX for locally advanced and borderline resectable pancreatic cancer. Ann Surg.

[19] Conroy T, Desseigne F, Ychou M, Bouche O, Guimbaud R, Becouarn Y (2011). FOLFIRINOX versus gemcitabine for metastatic pancreatic cancer. N Engl J Med.

[20] Sohal DP, Walsh RM, Ramanathan RK, Khorana AA (2014). Pancreatic adenocarcinoma: treating a systemic disease with systemic therapy. J Natl Cancer Inst.

[21] Dindo D, Demartines N, Clavien PA (2004). Classification of surgical complications: a new proposal with evaluation in a cohort of 6336 patients and results of a survey. Ann Surg.

[22] Bassi C, Dervenis C, Butturini G, Fingerhut A, Yeo C, Izbicki J (2005). Postoperative pancreatic fistula: an international study group (ISGPF) definition. Surgery.

[23] Wente MN, Bassi C, Dervenis C, Fingerhut A, Gouma DJ, Izbicki JR (2007). Delayed gastric emptying (DGE) after pancreatic surgery: a suggested definition by the international study Group of Pancreatic Surgery (ISGPS). Surgery.

[24] Wente MN, Veit JA, Bassi C, Dervenis C, Fingerhut A, Gouma DJ (2007). Postpancreatectomy hemorrhage (PPH): an international study Group of Pancreatic Surgery (ISGPS) definition. Surgery.

[25] Koch M, Garden OJ, Padbury R, Rahbari NN, Adam R, Capussotti L (2011). Bile leakage after hepatobiliary and pancreatic surgery: a definition and grading of severity by the international study Group of Liver Surgery. Surgery.

[26] Hoem D, Viste A (2012). Improving survival following surgery for pancreatic ductal adenocarcinoma--a ten-year experience. Eur J Surg Oncol.

[27] Neoptolemos JP, Palmer DH, Ghaneh P, Psarelli EE, Valle JW, Halloran CM (2017). Comparison of adjuvant gemcitabine and capecitabine with gemcitabine monotherapy in patients with resected pancreatic cancer (ESPAC-4): a multicentre, open-label, randomised, phase 3 trial. Lancet.

[28] Valle JW, Palmer D, Jackson R, Cox T, Neoptolemos JP, Ghaneh P (2014). Optimal duration and timing of adjuvant chemotherapy after definitive surgery for ductal adenocarcinoma of the pancreas: ongoing lessons from the ESPAC-3 study. J Clin Oncol.

[29] Tol JA, Gouma DJ, Bassi C, Dervenis C, Montorsi M, Adham M (2014). Definition of a standard lymphadenectomy in surgery for pancreatic ductal adenocarcinoma: a consensus statement by the international study group on pancreatic surgery (ISGPS). Surgery.

[30] Clavien PA, Barkun J, de Oliveira ML, Vauthey JN, Dindo D, Schulick RD (2009). The Clavien-Dindo classification of surgical complications: five-year experience. Ann Surg.

[31] Verbeke CS, Gladhaug IP (2016). Dissection of pancreatic resection specimens. Surg Pathol Clin.

[32] Verbeke C, Lohr M, Karlsson JS, Del Chiaro M (2015). Pathology reporting of pancreatic cancer following neoadjuvant therapy: challenges and uncertainties. Cancer Treat Rev.

[33] Washington K, Berlin J, Branton P, Burgart LJ, Carter DK, Compton CC (2016). Protocol for the examination of specimens from patients with carcinoma of the pancreas.

